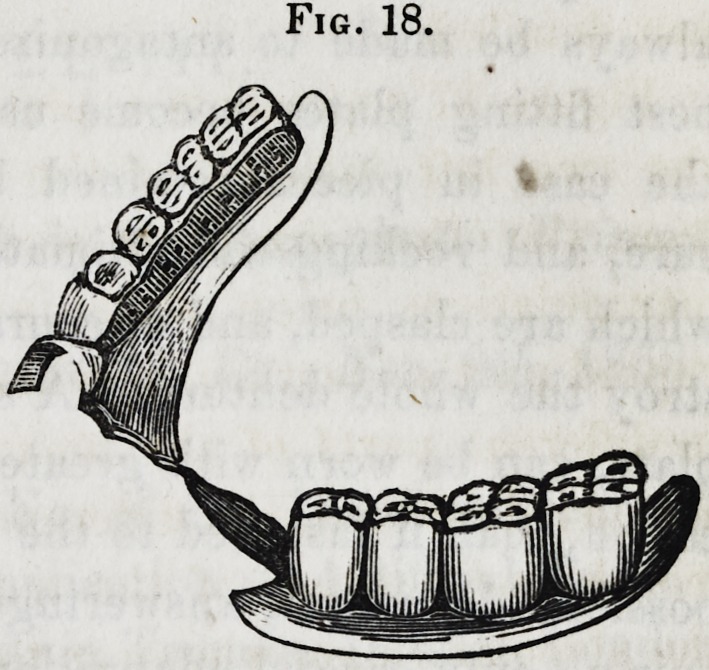# Mechanical Dentistry

**Published:** 1854-01

**Authors:** John Lewis

**Affiliations:** Buffalo, N.Y.


### ARTICLE II.

*Mechanical Dentistry*. By JOHN LEWIS, D. D. S., Buffalo, N.Y.

As the usefulness and beauty of artificial teeth depend on their being rightly constructed and properly adapted, it should be the object of the dentist in the application of a substitute for the natural organs, to secure the attainment of these ends; and with a view of aiding the young practitioner, I will give a description of my method of procedure, which, in some respects, is peculiarly my own; and in submitting it to the examination of the profession, I believe it will be generally approved; at any rate, I am willing it should be taken for what it is worth.

*Condition in which the mouth should be for the reception of artificial teeth.*—In the first place, it is necessary that the mouth and the gums should be in a proper condition to receive teeth; and as this is a subject of controversy, I will briefly explain what I consider a proper condition. In applying a full set, it is necessary that the gums should be in a healthy condition, the gums presenting no inequalities on the surface, especially if part of the teeth have been extracted a much longer time than the others; but if all or nearly all have been removed at the same time, an artificial set can be applied immediately, as I shall demonstrate in a subsequent part of this article; but not a permanent set.

Dentists who have practiced any length of time are aware, that it is often required and always desirable, to apply artificial teeth immediately after the extraction of the natural organs. It is desired on account of the alteration of voice, which almost invariably follows the loss of the teeth, as well as of the difficulty of mastication, to say nothing of the loss of beauty, and that pleasing expression of countenance which a beautiful set of teeth invariably give. Under such circumstances most persons requiring artificial teeth are willing to incur the additional expense of a temporary set, that can be worn without great inconvenience, until the mouth is in a proper condition to receive the permanent ones; and this is a matter easily accomplished, when done in the manner as hereafter described.

The dentist is often desired to furnish a dental substitute, when there is one, two, or more teeth remaining in the jaw; for example, the cuspid teeth, which very often remain, when all the others are gone, the practitioner contenting himself with a simple remonstrance, often consents to gratify the wishes of his patients, although well knowing, at the time, that such substitute will never fulfill the design for which it is intended, as well as it would if the natural teeth were all out. Such practice is reprehensible in the highest degree; such remaining teeth should be first extracted, then artificial teeth will be of much more use to the patient, and present a much better appearance in the mouth. It is seldom that artificial teeth applied without this precaution, can be worn any great length of time; for the one or two remaining natural teeth standing alone, with the artificial substitute, impinging upon them, soon loosen or become diseased, and have, ultimately, to be removed.

*Taking an Impression of the Mouth.*—When it is ascertained that the gums are in a condition to receive teeth, I proceed to take a cast or impression of the mouth. For this purpose, many dentists use plaster, but I prefer wax; and am decidedly of the opinion, that a much better impression can be taken with it than with any other substance now in common use; and it is evident, that wax is much more convenient for the operator, and more agreeable to the patient, than plaster. The cup or instrument to hold the wax, now in common use, is positively not fit for the purpose. It is impossible to take a good impression with it, without using so much wax, as to be very disagreeable to the patient. It is also unwieldy and unhandy; and with it, the operator is very liable to cut and lacerate the gums. With a little practice, and proper care, with those which I construct for the purpose, one can scarcely fail to obtain a correct impression; and as every dentist can easily make them for his own use, I will briefly describe the manner of constructing them. Take britannia metal of one-sixteenth of an inch in thickness, cut it to the required shape, then take a plaster model, cover it with wax to any desired shape or size; mould it in sand and get a metal model and counter-model, between which, stamp the britannia plate, in the manner as gum plate for teeth is stamped. Trim it, leaving it of a sufficient height, letting it run back as far as necessary in the roof of the mouth, then cut a wooden pattern for a handle, plain or otherwise, as suits the fancy, from which, cast a britannia duplicate, to be soldered to the front of the cup, for the better convenience of holding it while taking the impression. Of these, several different sizes are required for each jaw, so that one of suitable dimensions for any impression may be at hand.

FIG. 1.

Fig. 1, represents an impression cup for the upper jaw; made in the manner as just described. It should be as near the size of the alveolar border of which it is desired to take the impression as possible, then it will not require a redundancy of wax.

Figure 2, represents an impression cup for the lower jaw, which should be as near the size and shape of the particular jaw for which it is intended as possible. The figure represents a cup, suitable for taking an impression when the natural teeth are all gone, and the gums in a proper condition for receiving a substitute, but where part of the natural organs are remaining, the cup should be much deeper than is here represented. In such cases, it should be deep enough to cover the alveolar ridge without coming in contact with the remaining teeth.

FIG. 2.

The cup, before the wax is put in it, should be smeared with oil, so that the wax will leave it readily; and also move about in it easily. The wax should be made very warm, and worked in the hand until no lumps can be felt in it. Care should be taken not to put too much in the cup, for if there is more in it than is absolutely needed, it will protrude over the edges and be likely to have its shape altered while taking it out of the mouth, thereby spoiling the impression. The impression should be warmed before the wax is put in, to keep the latter soft and plastic while the impression is being taken. The wax, too, if oiled previously to use, will more readily adapt itself to the inequalities of the mouth, a desideratum of the utmost importance.

In models for parts of sets, it requires, as I have before said, deeper cups and more wax, or the teeth will strike the bottom of the cup, rendering it impossible to obtain a perfect impression. The operator can easily tell, on examining the mouth, if the cup is of the right size and depth, and the amount of wax required, before taking the impression. The operator should stand at the back and right of the patient while taking the impression, holding the cup firmly with the right hand, with the middle finger of the left opposite the centre of the roof of the mouth, if for a full set, or otherwise, according to the circumstances of the case. He should press it easily, but firmly on the jaw, until the alveolar ridge, and as much of the other parts of the mouth as is necessary, are completely imbedded in the wax; he should then remove it slowly and with great care, using the precaution not to move or alter the shape of the wax. The impression should then be carefully examined, and if it is not perfect in every part, the operation should be repeated.

Water should now be poured into the impression to expel the air from the indentations made by the teeth, or any other inequality. Now, take a piece of zinc or tin, about three inches wide, and as long as may be required to surround the impression, leaving a space of half an inch or more between the wax and cup and hoop, then take calcined plaster and mix it with water to about the consistence of batter, (Novia Scotia plaster is the best, as it sets harder and quicker than any other,) and pour it on the impression, filling it and the hoop. The plaster should be mixed as expeditiously as possible, in order to have it set quickly and firmly; and if necessary, the process may be expedited by mixing it in warm water. It will generally consolidate sufficiently in about five minutes, when the wax may be taken off and the model trimmed. If the model is for a full set, the wax can be taken off with a knife or other suitable instrument; but if there are teeth on the model, it should be placed in a proper vessel and the wax slowly melted from it; for, when separated in any other way, there is danger of breaking the teeth from the model. When the wax is removed the model should be trimmed, beveling from the gum outwards, so that it will draw easily from the sand.

*Manner of Obtaining a Metallic Model.*—The sand to be suitable for moulding, must have sufficient moisture in it to pack well, otherwise it will not work; if it is too dry, it will not draw, and if too wet, it will "blow." I use zinc for my models, as it is the best metal for that purpose, when pure, that I have ever used. I use lead for my counter-models, but care must be taken not to have it too hot; for if there should be a soft spot in the zinc model, they will fuse together. In order to make the plaster model draw from the sand easily, without bringing any of the sand with it, it should be brushed with powdered charcoal until the surface is perfectly dry, leaving it slightly covered with coal dust, when the sand is packed around it; this is absolutely necessary, especially when there are teeth on the model, or the sand will not draw from the interstices between and around the teeth. After the model is perfectly trimmed, I place it upon a small board in my box containing the sand, which has been sifted through a fine wire sieve, to remove any gravel or lumps which may have been in it, and then place a hoop of wood or tin (as may be convenient) around it. The hoop should be several inches in diameter, and three or four inches in height. I then pack this full of sand around the model, not packing it so hard but that it will yield a little by slightly tapping the model. I now place another board upon it and turn it over, and then carefully remove the model, after driving a sharp instrument of some kind into it, which enables me to do it more conveniently. Any loose sand which may have fallen into the mould can now be removed by holding it up and blowing into it. Melted zinc should now be poured into the mould, in as careful a manner as possible, pouring it into the back part, or wherever it is not desired to cover the model with the plate; for if poured directly upon such part of the model, it may displace some of the sand and spoil the model. After the zinc model becomes cold and hard, cover it with sand, and then turn it over, after placing the board upon it to keep the whole in its proper place; then remove the sand from the face of the model, and as far up the side as may be necessary to give it the requisite strength, using the precaution to remove the sand from the part representing the gum and the palatine surface, as well as from around the teeth, if there are any. If this is neglected, it will be more difficult to stamp the plate into its proper shape and secure a perfect adaptation. The melted lead may now be poured upon the zinc model, to which it will accurately adapt itself, forming a perfect counter-model, if the requisite care has been taken to have the lead of the right temperature; if it is too hot it will be likely to fuse the surface of the zinc, and unite the two so that they cannot be separated, or at least so as to spoil the model.

*Manner of Alloying Gold for Bases for Dental Substitutes —Thickness and Construction of Plates—Thickness of Standards or Backings for Artificial Teeth, how Shaped, and Solder most proper to be employed for Uniting them to the Base.*—It is, I believe, conceded by nearly all, that gold is preferable for a plate or base for artificial teeth, than any other metal, or at least for general practice. I know that platina is sometimes used and preferred, but it is very difficult to procure it, and cannot be worked to as good advantage. On this account the writer confines himself to the use of gold and silver. He would observe, however, that the manipulations in platina are substantially the same as will be described for the other named metals. Gold, in its pure state, or of the fineness of American coin, is too soft and pliable for a base; it is, therefore, necessary to alloy it with some metal to make it of the desired stiffness, and still leave it in a condition not to be acted upon by the fluids of the mouth. The question now is, what is the best alloy for that purpose? A great many have been used. My method of alloying gold is as follows: Take one part of copper and two of silver, and melt together. With four grains of this, alloy twenty-four grains of American gold. This produces a metal sufficiently pliable, and one which is in every respect just what we want for our purpose. The writer usually takes three cent pieces for his alloy, as these contain about the right relative proportions of copper and silver. A sufficient number of these to reduce the gold to the desired standard may be added to the coin, and the whole put in a common crucible, then add a little borax; this is necessary to make them fuse together in a proper manner. Place the crucible in a furnace, or if this is not practicable or at hand, a good hot charcoal fire will answer the purpose. It should remain in the fire until the gold comes to a white heat, when it may be removed and poured into an ingot mould of the proper size and shape. The writer uses ingot moulds of different sizes, according to the amount of metal he desires to cast, but all of them have the same thickness, say the sixteenth of an inch. They should be made of iron, and before pouring the metal in, the mould should be heated and well oiled with sweet oil, to secure a smooth ingot and leave the mould readily. If the metal is poured in when the mould is cold it will be chilled as soon as it comes in contact with it, and the ingot will not be of uniform dimensions, and when this is the case, the plate, when it is rolled down, will not be perfect. When it is desired to get out a small quantity of gold—say enough for one set of teeth—it can be done in a much shorter and easier way, and one which the writer greatly prefers, as a general thing, to the other.

FIG. 3.

Take a piece of pine charcoal and place it upon a soldering frame, such as is represented in Fig. 3, to which it is confined by wires passing around it, and through small holes in the edge. A cavity as large as may be necessary is scoped in the charcoal. In this place the gold and alloy, with enough borax to make it fuse together freely; another piece of coal is placed over this so as to cover about two-thirds of it over. The flame from a spirit lamp is directed upon the metal with a common blow-pipe, keeping up as regular a degree of heat as possible, until the metal melts and becomes white. Then remove the upper piece of charcoal and place the flat face of a heavy hammer upon the metal, letting it bear as heavily as may be required to make it of the desired thickness. In this manner the gold is not brought into as regular and uniform shape as in casting, it is true; but if care is taken in forming the cavity in which it is melted, it can be brought into very good shape; in fact, into almost any shape which may be desired; for, if the charcoal is clear and good, it will burn but very little, while melting the metal. The cavity in the coal should be made of an oblong shape.

A spirit lamp and common blow-pipe, when they can be used without injury to the lungs, are much to be preferred to any other soldering fixtures now in use. The lamp should be large enough to hold a half pint or more; the shape is immaterial, excepting that it should have a spout coming from the bottom, three or four inches long and three-fourths of an inch in diameter. The end of the spout should be on a level with the top of the lamp; and it should always be filled with the wick, or the flame will be liable to extend into the body of the lamp, setting fire to the fluid and cause an explosion, which, if not very dangerous, can be easily avoided by observing the above precaution.

FIG. 4.

FIG. 5.

Figure 4 represents a lamp of this kind, which, for shape and use, is as convenient and suitable for this purpose as any that can be employed. It has a small spout at letter *a*, for the better convenience of emptying it at night, or any other time when it is not in use; for if this is not done it cannot stand long without the alcohol evaporating. This lamp occupies but a very small space, and can be set on the table directly by the side of the operator, rendering it unnecessary for him to move to anneal the plate while stamping it up. It is low, and of such a shape that it is not easily upset, and in every respect is better adapted for a "mechanical" dentist than any other.

The blow-pipe should consist of a simple tube, gradually growing tapering towards the end, and curved as in figure 5. This can be made with a joint, so as to change the points; or it would be still better to have two or three blow-pipes, with different sized orifices; because it is necessary to have a larger orifice in melting down or in soldering a large piece of work, than in a small piece where it is desired to direct the flame, into a small aperture, as is often required in repairing a piece. If the blow-pipe is of brass, it should have a mouth piece of silver, to secure the lips, &c. from the peculiar effects which brass has upon them. With a lamp and blow-pipe of the description above given, one can execute a piece much quicker, better, and with less fatigue than with the "compound blow-pipe and lamp," or any of the "labor-saving machines" now in use.

After the gold has been brought into proper shape to reduce to plate, proceed to get it out, by passing it through a mill, such as is commonly used for this purpose by gold beaters and dentists generally. The rollers are of polished steel and very hard, and worked by a crank attached to each end, or by a single crank with cogs connecting the rollers; these are better for the use of one person, or in getting out a small piece of gold. The mill which the writer uses for this purpose, is made by J. Lodge & Co., Philadelphia, with cogs connecting the rollers, so that one person can work it. It can also be used with two cranks, when required for milling a large piece, where a greater amount of pressure is needed. The rollers should be kept at an equal distance from each other at both ends, and should be brought a little nearer together every time the gold is put through, by means of the set screws, at the top of the mill. The gold should be kept well annealed during the process of rolling or it will crack, and when the plate is of large size, it should be rolled backwards and forwards in the centre, several times, each time it goes through the mill; othewise the ends will become thinner than the centre, and when this is the case they are liable to split or crack. By following the above directions the plate will be kept of a uniform thickness throughout, unless the gold is of inferior quality, or has not been properly treated. In this case, it will be necessary to remelt it, and when this is done, if it should crack or be flawy, the ductility will be increased by throwing into the crucible, when the metal is fused, a little sal ammonia; if it should not then work well, a little nitre should be thrown into it when melting the next time. The desired result will often be more certainly obtained by throwing into the melted metal a little nitre and sal ammonia at the same time, but the remelting will not unfrequently remedy the difficulty.

For determining the thickness of the plate, a common wire guage with thirty-one holes may be used, rolling the metal down for a plate for a full upper set until it goes into number twenty-five. The thickness should be varied according to the size, shape, and condition of the alveolar arch. When the roof of the mouth is deep and the gums in a healthy and good condition, it may be thinner than this, but a thicker plate is often required, especially for parts of sets, and when the palatine arch is very shallow. For the lower jaw, a much narrower base being required to prevent it from springing, the plate should be thick enough to fit tightly in twenty-two, leaving the edges of the full thickness of the other parts of the base. The alveolar ridge and gum of the lower jaw are of such a shape as to render the application of a dental substitute here altogether more difficult than for the upper jaw. The mucous membrane and muscular attachments of the former are loose and liable to be moved by the motions of the jaws, and to displace the piece. This tendency is almost always felt, and cannot be overcome but by wearing the plate constantly in the mouth, until the gums become firm and the loose integuments cease to act upon it, which, if the adaptation is perfect, will ultimately be the case.

The writer has, in several cases, encountered obstacles in the upper jaw, where the action of folds of mucous membrane and muscular attachments, whenever the jaws or lips are moved, displaced the piece, and where the difficulty could only be overcome by cutting out portions of the plate. But it is impossible to point out all the difficulties and give specific directions with regard to the manner of overcoming each. It is impossible to lay down rules that will apply to every case, and hence, the judgment of the practitioner will oftentimes have to determine the means most proper to be employed.

The views of the patient, with regard to the manner in which the piece is to be constructed, is sometimes opposed to the experience and judgment of the dentist. For the purpose of having it as light as possible, he sometimes insists upon having the plate too thin to sustain the force to which dental substitutes are ordinarily subjected. To have the plate thick enough to secure the requisite strength, and prevent the edges from cutting the parts with which they are in contact, has long been a desideratum with mechanical dentists. Many ways of thickening the edge of the plate have been proposed and practiced; some cut a narrow strip of gold and solder it around it; others double or roll the edge over upon itself, but none of these methods answer a very good purpose. They have been tried, and, I believe, most of them abandoned. The writer has a better method, one which he has practiced for several years with complete success; and one which, when generally known, will, he thinks, be universally practiced. It consists in making a hollow wire, or small tube, with a section of it removed. Take a piece of pure gold or American coin, as it is required to be very ductile to get it into the requisite shape; cut a strip off of a five dollar gold piece, about one-sixteenth of an inch in width; this by a little hammering will become a wire of two or three inches in length, which draw through a common wire plate, making it smaller and smaller, keeping it well annealed during the operation, until it will go into number eighteen of the wire guage; then anneal the wire, and run it through the rolling mill, flattening it down to number twenty-seven; it is then a uniform strip of gold, between one-eighth and one-sixteenth of an inch in width. This done, take a sufficiently long piece of steel wire, about number twenty-five, around which double the end of the gold strip, and then draw the two through a large hole in the wire plate, the strip partially wraps itself around the wire; this is continued through successively smaller holes in the plate, until it will pass number eighteen. The steel wire can then be removed, through the opening between the edges. The gold wire may now be drawn through smaller and smaller holes until the edge of the plate fits tightly into the groove in the wire. The latter is then annealed, to make it bend easily to the inequalities of the plate, to every part of the edge of which, after it has been properly fitted and reduced to the right size, it should be accurately adjusted. This done, it is confined with small annealed iron wire, and then soldered. But for this, very little solder will be required if the wire is properly made and applied, as it will fit almost perfectly the edge of the plate. A grooved wire soldered around the edge of a plate makes a base very strong and durable, and capable of receiving a beautiful finish, besides, it is lighter; as the plate can be made as thin as number twenty-seven, and still be stronger and less liable to spring than a plate of the thickness of number twenty-five in guage-plate without the binding.

The gold plate for the standards or backings to the teeth, should be as thick as number twenty-three, to be of sufficient strength to support the strain which comes upon the teeth. Each backing, however, should be beveled off towards the approximal and cutting edges of the tooth at the base, where it is to be soldered to the plate it should be left the full thickness.

In the majority of cases, the standards are so light that they are continually breaking, and then, in mending them, others are melted and the teeth broken, to the great injury of the entire piece; for there is always liability of injury to a set of teeth to subject them, after having been worn, to a sufficiently high degree of heat to melt solder, especially, if the solder is of the requisite fineness. It is partly for this reason, that the writer prefers and applies standards to teeth, in the manner to be hereafter described.

The solder should be of a quality to flow at a considerable lower temperature than is required to melt the plate, and still be fine enough as to prevent being acted upon by the fluids of the mouth. In making solder, the writer uses the scraps cut from his plate, as he always knows the quality of the gold used for this; his solder is composed of one part brass spelter and seven of gold scraps fused together. He then rolls it down as thin as number twenty-four, and cuts it into short strips, it being more conveniently placed in this form than any other; and it should always be the object of the dentist to use as little solder as possible, as this is more liable to be acted upon by the saliva, if it is not very pure. For silver solder the writer uses one part of spelter to four of silver, melted together, and made into the proper shape for convenient use, using no more, however, than is necessary to put the work together in a permanent and substantial manner.

*Manner of Shaping and Stamping up a Plate.*—Having premised thus much, I will proceed to describe the method of procedure which I pursue in getting up a plate. Take a piece of paper, and cut a pattern which will cover such portions of the model as it is desired that the plate should occupy. Some dentists use sheet lead for this purpose, but it is immaterial, so that a correct pattern is made. I prefer paper, because it can be pressed into all the inequalities of the model, and again straightened out without altering in the least its size and proportion; lay this pattern upon the gold, and with some sharp pointed instrument, trace the outlines of it upon the plate, then with a heavy pair of shears, or snips, as they are commonly called, cut out the piece of plate so marked, and after annealing, bend and adjust it with a hammer, and different shaped plate pliers, as near as possible to the metal model. Now place it between the metal model and counter-model, and with a heavy hammer, proceed to stamp it into shape. It will be better to remove it now and then, to ascertain if it retains the right place; and by cutting here a little, and bending there a little, it will be swaged into its proper place and shape, without danger of cracking. It should be annealed several times during the process of stamping. If this is not done it will be liable to spring and rock upon the model in soldering the standards to it. The cavity should be formed in stamping the plate, and this is done in the following manner. Take a piece of card board, and cut it into the desired shape and size for the cavity; of these a sufficient number may be cut to make the cavity as deep as may be necessary. Each succeeding one should be cut somewhat smaller than the preceding one, and then placed upon the model as represented in Fig. 6, with the first or largest one next to it. A thin coating of wax is placed between every two, as also on the side of the whole next to the model, to make them adhere to it. These are placed upon the metallic model after the plate has been partially swaged, but before it has assumed its proper shape. Thus adjusted, the process of stamping is completed with a few blows of a heavy hammer. A cavity formed in this manner, is as easily made, and answers every purpose equally as well, if not better, than those commonly used. Different methods have been adopted for forming a cavity. It is sometimes done by placing a piece, of the right size, shape and thickness, of wax on the plaster model, at the place required for the cavity. But the first method is preferred by the writer, as it leaves the impression and model in the precise shape of the mouth; and this is sometimes necessary, for we often wish to know the exact shape of the mouth, where the cavity is placed. Some suppose, that the cavity is formed expressly for the purpose of a vacuum to hold the plate up and in its place; but this is not the only object to be fulfilled by it, it keeps the plate from riding upon the roof of the mouth, when the palatine mucous membrane is inflamed and in a thickened condition. But after the irritation, sometimes occasioned by the plate when first applied, subsides, it seldom happens that there is any trouble

FIG. 6.

in keeping it up, even if there be no cavity in the plate. The truth of this observation has been verified by the writer. He has noticed, that where small cavities had been formed in the plate on the inside of the teeth, the slight swelling of the mucous membrane of the palate render them useless, and caused the plate to drop. In similar cases, with a cavity formed in the manner as described, there has been no inconvenience, unless the swelling has been very considerable. I am satisfied, however, that the cavity is not required to keep the plate in its place; for I have observed that it fills entirely up with loose spongy structure, caused by the tendency of the mucous membrane to fill the vacuum, without causing the least trouble or inconvenience to the patient; as in every case it kept its place equally well, although it is not to be denied, but that the cavity is sometimes a help in keeping it up; especially when the piece is first applied, and this is the time it is most needed.

While stamping the plate, it should be trimmed to the proper size and shape, letting it extend back as far as practicable, so that it can be worn with ease and comfort by the patient. It should be scalloped out in front at the median line to admit the frænum of the lip, covering the alveolar ridge, exteriorly, as far as the gums extend or the case will permit. If it is desired to bind the plate, it should now be done, adjusting the hollow wire upon the outer edge, and soldering as before directed. The wire should not be applied to the edge of the plate across the plate, as it would be unpleasant to the patient, and it is not required to give strength to the base. The wire having been put on and soldered, the plate should be again swaged to restore its perfect adaptation to the model.

If the patient is at hand, or it is thought necessary, the plate should now be tried in the mouth, to ascertain whether its adaptation is perfect, for, if it is not, the different operations will all have to be repeated; but this is so seldom necessary that the experienced practitioner may proceed at once to attach the front standard to its place, before trying the plate in the mouth.

The base is now ready to receive the teeth, or rather the backings, as in the writer's method of constructing dental substitutes, these are all secured to the plate before the teeth are attached. His manner of doing this, and applying the teeth, constitutes what he believes to be an important improvement upon the methods usually practiced. It is as follows: Take a strip of gold, of the width required by the length of the teeth, and long enough to support the incisors and cuspidati, the exact length is not required, as it can be cut off after the teeth are fitted, nor is the exact width material, although it would be better to be somewhat wider than is necessary, so that it can be trimmed when tried into the mouth. The proper length of the teeth can also be determined by the relative length of the standard, lips, &c., in connection with the general physiognomy and appearance of the face. The standard or backing for the six front teeth should be, as before stated, as thick as number twenty-three in the guage plate, to give the necessary strength and durability to the teeth. It may afterwards be beveled off to the teeth without thinning the whole and rendering it liable to break.

FIG. 7.

The front standard, when first cut out, should generally represent a segment of a six inch circle, such as is shown in figure 7. The size of this circle varies in different jaws; in some it is much smaller and in others larger. This should now be bent into the segment of the circle required for the teeth, and the size of which may be determined by the general shape of the jaw and face, and the concave edge filed to fit the inequalities of the alveolar ridge, at the particular place it is desired to attach it to the plate.

FIG. 8.

FIG. 9.

The appearance of the standard is now represented by figure

8, showing it bent and filed to fit the model represented in figure 6.

The operator should carefully observe, while taking the impression and trying the plate, the position and prominence of the gums, and each place on the plate it is necessary to set the teeth. By practice and careful attention, he can, in this way, ascertain by the eye the proper place on the plate for the arrangement of the standard, which will seldom be incorrect. The standard should now be placed in the required position, and securely bound there with small annealed iron wire; it can then be barely attached on to the plate, with a small piece of solder, when the wire should be removed and the plate again tried into the mouth to ascertain if it is in the position required. If there are natural teeth in the lower jaw, these will determine the position, as the standard should shut over the lower teeth, when the jaws come together in the natural way; but if there are no front teeth in the inferior maxillary, its place should be determined by the relative position of the two jaws, taking into the account the restoration of the contour of the face and lips, making suitable allowance for the thickness of the teeth on the outside of the standard. If the standard is found to be too far out, or too far in, or in any way different from what it should be, it should now be broken from the plate, with a pair of pliers; or if it is so firmly attached as to endanger the plate, it can be placed upon the charcoal and the solder melted, when it can be removed with a slight blow with the end of the blowpipe. The standard should now be readjusted, and fastened as before, with solder. The plate should then be again put into the mouth, and the standard having been adjusted to it, in its right position, it should be trimmed to the required length. The median line or centre should now be marked on it with a sharp instrument, to designate the proper place for the central incisors.

The plate may now be removed from the mouth and a notch filed in the standard at the point thus marked, so that if the former should be obliterated in heating the plate, no mistake will be made in adjusting the teeth. The standard should now be firmly bound in its place with iron wire to prevent the liability of its being moved, and then permanently soldered to the plate.

*Soldering the Standard to the Plate.*—The standard having been properly adjusted, borax ground with water should be placed along the line of union, and this can be done best with a camel's-hair pencil. It should be of the consistence of cream, and applied wherever it is desired the solder should flow. If a sufficient quantity is not applied, the solder will not flow freely but form itself into a globule where it is placed, or will simply adhere in a body to the plate. No more solder should be employed than is necessary to unite the backing securely to the plate. The standard having been filed down to the length required for the teeth, the latter may be selected, taking care to have them correspond in shape with the natural organs, and the complexion, and general contour of the face as nearly as possible. If they do not do this they may disfigure, instead of enhancing the beauty of the patient.

The teeth are adjusted to the standard, beginning with the central incisors, by punching holes in it to correspond with the rivets in their inner or palatine surface, and for this purpose a common dentist's punch is used; but it will require some practice to make them in the proper place. The best manner of doing it is to place the tooth upon the plate and standard, as near in the position it should be as possible, and then press it firmly against the latter. This will leave slight marks indicating the place for the rivets. Slight indentations may now be made at the places thus marked with the punch, and the tooth again tried to ascertain if they are in the right place; if so, they can now be punched through and the inside counter-sunk, so as to form a place in which to make a head to the rivet. To do this, a suitable instrument may be used. In adjusting the central incisors, a very narrow space may be left between them, or their approximal surfaces may barely touch, but but care must be taken that they do not crowd each other, and the base of the teeth should be ground until they fit the plate. The lateral incisors should next be adjusted, and these should be a very little shorter than the centrals, and the cuspids slightly longer, about half the length of the cusp on the end. But as yet the teeth are not fastened, the holes only having been punched and the teeth fitted. Each one, however, will keep its proper position, if the holes are not made too large, a thing which should be carefully avoided. If this is neglected the teeth cannot be securely fastened to the standard. The incisor and cuspid teeth having been accurately fitted, the ends of the standard, back of the last mentioned teeth, should be filed off.

FIG. 10.

The teeth may now be taken off, when the plate and standard will present the appearance shown in figure 10. The next thing to be done is to fit the standards to the base for the bicuspid and molar teeth. A strip of gold, equal in width to the length of the teeth, and of the right thickness. The end is then bent to nearly a right angle, and filed to represent a long triangle, with the point towards the plate. Thus shaped, it will present the appearance as represented in figure 11.

FIG 11.

FIG. 12.

This standard will set perpendicular on the plate, and the bent extremity will fit the end of the front standard, which inclines outwardly. The standard should now be bent, forming a slight curve as far back as the first molar, and the edge filed to fit the inequalities of the plate. If it is required, by the difference in the thickness of the molars and bicuspids, there should be another slight, but abrupt curve made in it between the second bicuspid and first molar, which sets in a little farther on the plate than the former. The triangular bend of the standard should be made as wide as may be required by the thickness of the teeth.

The standard is now ready to solder to the plate, and its appearance is represented by figure 12. It must first, however, be bound in its proper place, with iron wire, in the manner as before described. The line of union between it and the standard should be first soldered. This done, the standard for the teeth on the other side of the plate should be made, and in like manner soldered; and during this process, if there should be any hole in the front standard, it may at the same time be closed, by flowing a piece of solder into it, when another may be made, using the precaution to punch it in the right place. Each of the side standards should be sufficiently long to support two bicuspid and two molar teeth—this being the number usually employed in an artificial denture, though one less than occurs in a natural set. The alveolar arch, however, contracts after the loss of the natural organs, so that in their replacement a less number are required. The standards having been soldered to the plate, the piece should be boiled in sulphuric acid, diluted with five parts of water to one of acid, to remove the borax, or any other foreign matter which may have collected upon it, during the different solderings and other manipulations. This may be done by placing it with the diluted acid in a copper ladle, and holding it over a spirit lamp until the acid boils, using the precaution that there be no other metal in the ladle with the plate, especially iron, as this would be precipitated upon the gold. If, therefore, the solder has flowed upon the iron wire used for binding the strips of backing to the plate, it should be first carefully removed.

The front standard should now be filed out between the teeth, cutting it down about half their length, and then rounding each separate point behind the tooth into a sort of gothic arch, leaving it as high as may be considered necessary, to secure the requisite strength, and beveling the inner edge down to the tooth. In doing this they should be made to resemble, as nearly as possible, the inner surfaces of the natural teeth.

At this stage of the operation the piece will present the appearance as represented in figure 13. The outside of each standard should now be filed to form a flat surface, on which to fit the tooth, for, if it is left rounded, it will be difficult to rivet

FIG. 13.

the teeth in such a way to prevent them from rocking, which, if not guarded against, would cause the rivets, in a short time, to break. Each of the six front teeth may be tried on separately, and if they do not fit accurately, they should be ground until they do. If they crowd each other, they may be ground a little on their sides also, so that they will stand entirely separate from each other; or at any rate, so as not to press one against the other, as this would be likely to cause them to break while riveting to the standard. For grinding, a small corundum wheel, turned in a light foot-lathe, may be employed, and with an apparatus of this sort every resident dentist should be provided.

After the front teeth are fitted, they may be riveted to the standard. This is done by placing the tooth on the lead model, and hammering the end of the rivets into the hole countersunk for that purpose, forming a head on the rivet, in such a shape that it can be burnished down smooth with the standard. For riveting, a very small light hammer, with one end shaped like a wedge, similar to the riveting hammer used for other purposes, but with the riveting end longer than usual. When the front teeth are all secured in the manner as here described, the piece should be again tried into the mouth, and the back standards trimmed to their proper length. If there are teeth in the lower jaw, the patient should be kept at hand, so as to repeatedly try the work into the mouth, to enable the dentist to select such as are of the right length and shape; but if lower teeth are to be supplied, he should be governed in this matter by symmetry and proper proportions, antagonizing those of the lower jaw with these. The bicuspid and molar teeth may be a shade darker than the incisors and cuspids. The second should be a little larger than the first.

The holes for the rivets of the molar and bicuspid teeth may now be punched and countersunk, as in the manner as before described; and the teeth when properly fitted, riveted to the standards, and the heads properly burnished. When all are properly secured, the standards should be filed off even with the inner edges of the teeth, and as far down between them as possible. But previous to this, the plate and standards should be made as smooth as possible, removing all superfluous solder with scrapers and gravers of different shapes and sizes, preparatory to polishing, the manner of doing which will hereafter be described.

Fig. 14.

Figure 14 represents a piece at the foregoing described stage of the operation, the molar and bicuspid teeth, however, being of a somewhat different shape from those represented in the cut which was taken from a set made as here described, but with teeth manufactured some years ago; it being only intended to show the manner of mounting.

In the construction of a dental substitute for the lower jaw, a somewhat different method of procedure is adopted, the manipulations however being principally the same. In the first place, the plate should be much thicker than that for the upper jaw, fitting tight into about number twenty-two of the plate guage, if its edges are not to be bound with hollow wire, and the advisability of doing this should depend in a great measure, in fact entirely, on the shape of the parts upon which it is to rest. When the alveolar ridge is very flat and narrow, a thicker plate will be required to sustain the pressure which will naturally come upon it, without springing or bending.

In getting out a base for a lower set, a paper is cut and fitted to the model, which serves as a guide in cutting out the plate. This last is annealed and swaged as before described, repeating the operation of annealing and swaging until it fits accurately the model. The plate for a lower set being much thicker and heavier than is required for an upper denture, it will be more difficult to stamp into its proper shape, but if it be frequently and well annealed, it may be adapted perfectly to all the inequalities of the parts. But previously to swaging it should be partially fitted to the metallic model with plate forceps and pliers, and during the process of stamping, it should, from time to time be trimmed until it is reduced to its proper dimensions, and in such a way that the edges shall not rest too firmly on the folds of mucous membrane on either side of the alveolar ridge. The ends of the plate should extend, as a general thing, a short distance upon the coronoid processes.

If there is the least apprehension that the model is not perfect, the plate should now be adjusted to the mouth. When it fits perfectly, the front standard, which usually consists of a straight strip of plate, with the edge filed to fit the inequalities of the base, may be soldered at one or two points, in nearly a perpendicular position instead of projecting as for the upper front teeth, and as nearly in its proper place as can be ascertained previously to being tried in the mouth. When the plate is placed in the mouth, it should shut about one-sixteenth of an inch on the inside of the upper standard, so as to give room for the under teeth on the outside of it. It may now be cut to the right length, and the median line marked upon it. This done, it may be soldered permanently to the plate, unless it should be found necessary to alter its position. In this case, it should be first removed and properly adjusted in the manner as before described. The incisor and cuspid teeth may now be fitted to the plate, and the ends of the standard cut off on a line with their posterior approximal surface. The next thing to be done is to prepare and attach the standards for the bicuspid and molar teeth in the manner as described for the corresponding teeth of the upper jaw, but which as a general thing require to be longer and placed more on the inside of the alveolar ridge, in order to make the teeth antagonize in a proper manner with those of the upper jaw. It is sometimes necessary to have the inner edge of the lower molars to stand in farther than the inner edge of the plate, and when this is the case, the lower edge of the standard must be bent outwards to make it meet the base. When the standards are soldered to the plate, the piece should be boiled in acid, as before described.

When the holes for the rivets in the teeth are punched, the standards may be cut down between the teeth, and beveled on the inside. The teeth should now be fitted and riveted to the standards, using the precaution in their selection to have them of the proper shape, size and color. During this part of the operation, the patient should be present, that the piece may, from time to time, be tried in the mouth, so that they may all be properly antagonized. The teeth being riveted, the piece may be finished in the manner as already described.

Fig. 15.

Figure 15 represents a dental substitute for the lower jaw, made in the manner as just detailed. It will be observed, that the front teeth incline a little inwards, it being necessary that they should do so, to bring their edges into their natural position; that is, on the inside of those of the upper jaw.

When it is necessary to have artificial gums, single gum teeth, or block teeth, with artificial gums, may be employed.

A fusible silicious cement, uniting single porcelain teeth to each other and to the base, covered with gum enamel, has, within the last few years, been introduced, to some extent, into practice, but the cement, unless the piece is made very clumsy, and too heavy to be worn with comfort, is liable, by the springing of the plate, to crack and scale off. But gum teeth are only required in those cases where it is necessary to supply the loss of the alveolar ridge, and unless called for, for this purpose, no advantage can be derived from them, therefore, when such loss has not taken place, single teeth are preferable to any substitute encumbered with a representation of gums. When applied under other circumstances, they disfigure rather than improve the appearance of the mouth and face, and are not worn with so much comfort and satisfaction as a denture composed of single teeth. Viewing the matter in this light, I never construct a dental substitute with artificial gums except for cases such as just described.

*Construction of Temporary Dental Substitutes.*—It often becomes necessary to replace the loss of the natural teeth immediately after their removal, with a substitute that can be worn until the gums and alveolar ridge are restored to health. In doing this, the method of procedure which the writer adopts, is as follows: Take an impression of the mouth with wax made so soft that it will not alter the shape of the gums or cause the patient much pain. If the wax is properly softened, and the impression taken carefully, it can be done as soon as the teeth are extracted, with little inconvenience and pain to the patient.

A perfect impression having been taken, water should be poured into it to expel the air. A plaster model is then taken, and the depressions representing the places from which the teeth were extracted, are filled with wax nearly to a level with the surrounding parts. If there should be other places in the gums represented in the model, from which part of the teeth had been previously extracted, fill them with wax so as to enlarge the model at this place, bringing it into as good shape as possible, for the permanent set. This done, procure the metallic models; after which, remove the wax from the plaster model, which is an exact representation of the alveolar ridge, and to which, after the plate is stamped, it can be bent to fit it, thereby improving its adaptation. The process of getting out a temporary plate is the same as one for a permanent set, except that it should not extend over the alveolar ridge in front any farther than is necessary to serve as a support for the teeth. The outer edge of that portion, upon which the incisors, cuspidati and bicuspids are placed, should not extend beyond these teeth, and in some cases, the writer has found it necessary to cut it off even with the molars. The reason for this is, that the inside of the gums never alter, and the outside, by pressure, can be made to assume almost any shape which may be desired. This is owing to the fact that, in the superior maxillary the alveolar ridge on the inside is principally composed of osseous structure, covered only with mucous membrane; and after it is fully developed, it undergoes but little alteration, while the alveolar border, after the loss of the teeth, is gradually absorbed, diminishing very considerably in depth and exterior dimensions, and is covered by more soft tissue, capable of adapting itself readily to a vacuum formed over it, and this, if long continued, will remain permanent. In view of this, the author, in adapting a plate, makes it to fit as perfectly as possible on the inside and on the ridge, in order that the gums may assume a good and regular shape. He also makes the air-cavity beneath the roof of the mouth somewhat deeper than in a permanent piece, as the entire plate will rise a very little.

Having properly adjusted the plate, the standards and teeth are attached in the same manner as for a permanent set, making them a little longer than would be proper at first, allowing for the shrinkage of the alveolar ridge. The outer edge of the plate is filed off close to the teeth, as far back as the second bicuspids, so that it may not be seen when the mouth is opened, permitting the remainder to cover the outside of the ridge. In a permanent piece it is sometimes necessary to cut the outer edge of the plate off in front of the incisor, cuspid and first bicuspid teeth, to prevent it from being seen when the upper lip is raised in talking or laughing. There are some cases, however, in which the piece cannot be made to adhere sufficiently tight without covering the entire ridge, and when, from peculiar conformation of the jaw, this happens, the stability and utility of the substitute should not be sacrificed to mere appearance. Teeth applied in this manner should be examined occasionally, and as the alveolar ridge absorbs, the plate should, from time to time, be bent, readjusting the outer edge to the altered shape of the gums.

The writer has, for a long time, been in the habit of supplying temporary pieces in the manner as here described, and many of which are still worn with comfort, and answer the purpose for which they were designed so fully, that the patients consider it unnecessary and refuse to have them replaced, thus showing the advantage and efficiency of this method of supplying temporary dentures, and even in cases where the gums were very ragged and irregular when they were applied. But as a general rule, temporary pieces applied in this or any other way, cannot be made to retain their adaptation after the absorption of the alveolar processes is completed; the nature of the change is such, in most cases, that pressure on one side will loosen and displace the piece on the other, and besides, the teeth, after a while become too short; but the wearing of a temporary substitute always has the effect of assisting the alveolar ridge to acquire a proper condition for the reception of the permanent piece.

Where part of the teeth have been out a long time, and the gums and alveolar processes around the remaining organs are, as is often the case, very prominent, it is scarcely possible to apply a substitute immediately after the extraction of these. The absorption of the prominent parts would soon cause the plate to lose its adaptation and press unequally upon the parts with which it is in contact, and as a consequence, some of the teeth would be forced out of place. Still, the loss of three or four from different parts of the mouth, no matter how long they may have been out, would interfere but little, if at all, with the application of a substitute immediately after the extraction of the remainder, as the places of the teeth, previously removed, may be built up with wax on the model as before described, and this will aid the gums to acquire a better shape than they otherwise would.

There are other cases in which the application of artificial teeth immediately after the extraction of the natural organs would be manifestly improper, as for example, when the gums are in an inflamed and swollen condition. The plate, in a case of this sort, after the swelling had subsided, would lose its adaptation and could not be worn with comfort, if at all.

*Parts of Sets of Artificial Teeth.*—The writer will now give his method of applying artificial teeth for either jaw, when only a part of a set is required. The cases, however, of this kind, are so numerous and varied, that it would be impossible to describe the exact method of procedure required for every case which is liable to come up in practice, as scarcely any two are precisely alike. The following description, however, will enable the practitioner to devise a substitute for supplying the loss of part of the teeth in any case which may occur.

It is scarcely necessary to premise, that unless the wax impression and models are perfect, it will be impossible to replace the loss of any of the teeth in such a manner as to subserve a valuable purpose.

In making a substitute for the upper incisors, a perfect model having been obtained, a paper pattern of the proper size, fitted to the natural teeth as far back as the first molars, or as far as may be considered necessary to give it the requisite surface, and covering the alveolar ridge in front, is made. I then cut my plate by this, and stamp it to fit the model, forming a large air-cavity in the palatine portion as for a plate for an entire set. The plate should then be stamped and fitted around the remaining teeth at their junction with the gums; this is said to be very objectionable by some authors, "because it will irritate and inflame the apices of the gum," but if the plate is made a very little larger than is required to cover the gum, so that when it is properly adapted, the edges will turn down a little on the teeth, it will obviate this objection, and make a much stronger and better plate than can otherwise be obtained. To do this in a proper manner, the plate should be cut considerably larger than is required, and swaged up against the teeth and afterwards filed off, leaving the edge slightly turned down on the teeth, as before described. A backing is now cut, sufficiently long to reach from one cuspid tooth to the other, and fitted to the inequalities of the plate; it is then slightly united to it with solder, giving it such an inclination, as that when the teeth are fitted on the outside, they will fill out the arch in a proper manner. The piece should now be placed in the mouth to ascertain if it fits perfectly, and also if the backing is in the right position; if so, it can be filed to the requisite length, and when taken from the mouth it should be firmly soldered to the plate. The piece should now be boiled in acid, and the backing cut down between the teeth to about one-half their length, or so as not to be seen from the outside. This done, the teeth may be fitted to the plate and backing, and firmly riveted; after which, the plate should be filed off on the outside, up to the teeth, and the edges made smooth, removing, at the same time, all superfluous solder preparatory to polishing.

FIG. 16.

FIG. 17.

Figure 16 represents a plate like the one just described, after it is ready for the reception of the teeth. It is not necessary to describe the method of procedure in making a substitute for any of the other teeth, it being nearly the same in all as that just detailed.

Figure 17 represents a plate with the standard attached for the two left incisors of the upper jaw. It varies but little from the other, except in size and position in the mouth, the manipulations being alike in each. In applying an interrupted partial denture, or where the teeth are scattered around in different parts of the mouth, the procedure is the same as already described, the difference consisting in the shape of the mouth and position of the teeth, the plate being carried over the alveolar ridge, wherever teeth may be required, far enough to serve as a support for the artificial teeth when placed in the vacant spaces where they are required.

When a substitute for the bicuspids on each side is required, or the bicuspids and molars, it is necessary to let the plate cover the whole anterior portion of the roof of the mouth; but cases of this sort are of rare occurrence, especially for the upper jaw, but in the lower they are less rare; and when such substitute is required here, a plate is constructed to fit the back part of the alveolar ridge on each side, extending it back as far as may be necessary, with a narrow strip running across the mouth, immediately behind the front teeth, perfectly adapted to the gums, which should be made somewhat thicker than is necessary for the other parts of the plate, and this can be done by rolling each end of the plate in the mill, after the whole is of the thickness required for the strip, then cutting out the base with the strip from the plate as thus prepared. Greater thickness for the part passing behind the front teeth is necessary, to give to it the requisite strength. The upper edge of this strip should extend up on the teeth a short distance, as also the plate where it fits around the posterior side of the last remaining tooth on each side, to preclude the possibility of "irritating" or "inflaming" the gum, and to give to the piece additional stability, serving as a sort of clasp. But this is not necessary when the plate can be kept securely in place without it, for it is certainly, in some degree, productive of injury to the teeth which support it. The standards and teeth are all fitted and attached to the plate, in the same manner as for a full set.

FIG. 18.

Figure 18 represents a substitute of this description, the six front teeth being still in the jaw.

It is scarcely necessary to say that the plate should be made perfectly smooth, to prevent it from irritating or abrading the gums and folds of mucous membrane, with which it comes in contact.

For finishing a piece, the writer uses flour of emery with olive oil, on pieces of pine wood, so shaped as that they may be applied readily to all of the irregularities of the plate. The wood being soft the surface is soon filled with the emery, by which the asperities and rough parts may, in a short time, be cut away. When this is done, the piece may be put on a small brush wheel, such as are commonly used for polishing, holding the surface of the plate, whenever required, upon the face of the brush, previously charged with oil and emery. The piece should now be thoroughly cleansed, after which, if it is desired, a beautiful finish can be given to it by using burnishers of dif-

19*

ferent sizes and shapes, fitted to reach every part of the piece. A fine finish can be obtained by using the ordinary brush and chalk that is free from small stones.

The piece is now ready to be introduced into the mouth, which being done, those portions which may not perfectly fit the gums should be bent to do so with a pair of pliers, as also any part which may press so hard upon them as to cause pain, or in any way prevent the perfect adaptation of the plate. The operator should also cause the patient to close his teeth, and those teeth which may strike before the others should be ground off, so that both rows of teeth may strike at the same time when closing the jaws; this is necessary, for if the teeth should strike sooner in one place than in another, it will loosen the plate, and cause the patient much trouble and inconvenience, if it be even possible to wear them at all. Artificial teeth should always be made to antagonize perfectly, for without this, the best fitting plates become useless failures, this is particularly the case in pieces retained by clasps, as the unequal pressure, and rocking will ultimately loosen and destroy the teeth which are clasped, and of course, if continued will certainly destroy the whole denture. A single tooth attached to a suction plate, can be worn with greater comfort, a week after its application, than if fastened to the other teeth; and it can be of no possible detriment, answering every purpose equally well, requiring only careful adaptation of the plate, as well as accurate antagonism with the other teeth. It is greatly to be hoped, that this method will eventually supersede all others, possessing as it does, so many advantages.

The writer has now described his method, of attaching artificial teeth to a continuous backing, and the advantage of this kind of backing over separate standards, as commonly used, are, first, increase of strength, for it will be readily perceived, that when biting upon one tooth, the strain is in part exerted upon all the rest; and, secondly, the continuous standard imparts additional strength to the plate; for with this band passing along and attached to it, it is almost impossible for the plate to bend or even spring, while separate backings afford no support to the plate whatever; and again, by means of a continuous standard with the teeth riveted to it, we avoid the necessity of heating the piece after the teeth are put to it, which is always productive of injury to them. In other words, it renders them less capable of sustaining the pressure to which artificial teeth are, as a general thing, more or less exposed. Besides, in the process of soldering, they are often cracked or broken.

I have now, in as few words as possible, described the method of procedure which I adopt in mounting artificial teeth, and I am disposed to believe, if fairly tested, it will be found superior to those usually practiced.

## Figures and Tables

**Fig. 1. f1:**
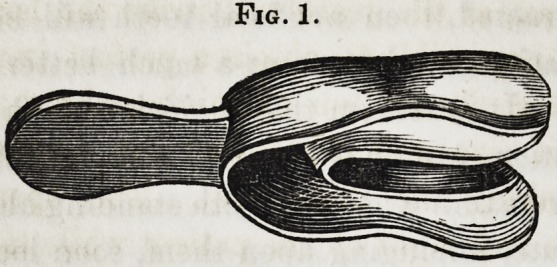


**Fig. 2. f2:**
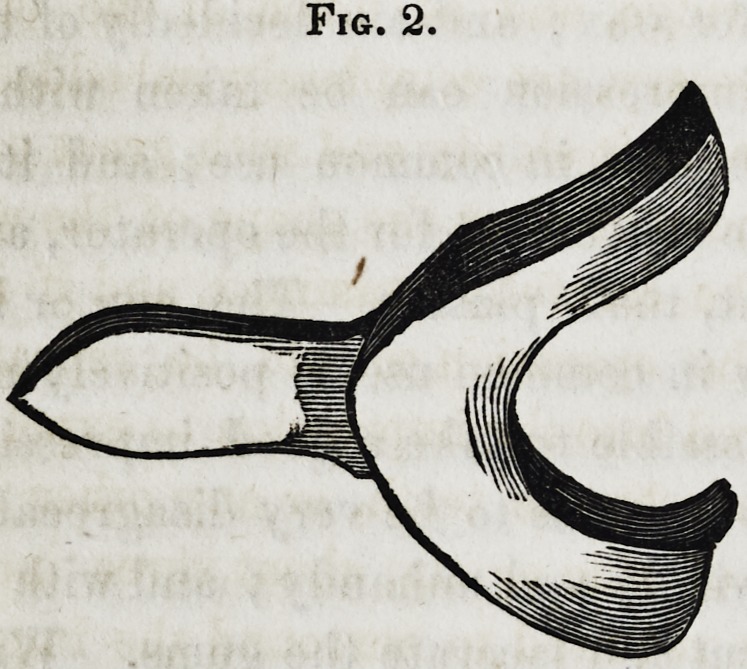


**Fig. 3. f3:**
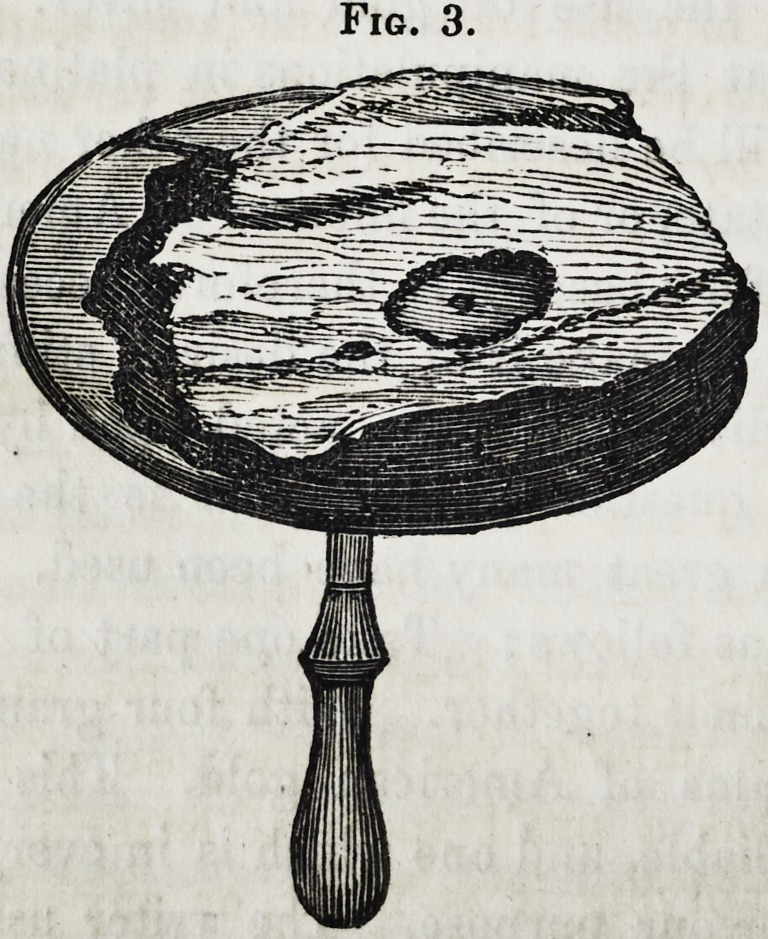


**Fig. 4. f4:**
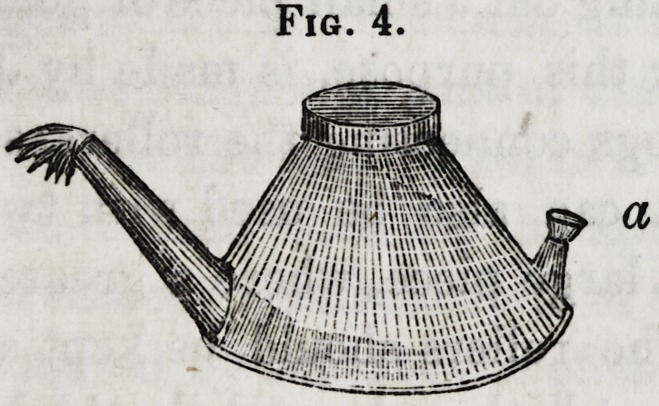


**Fig. 5. f5:**
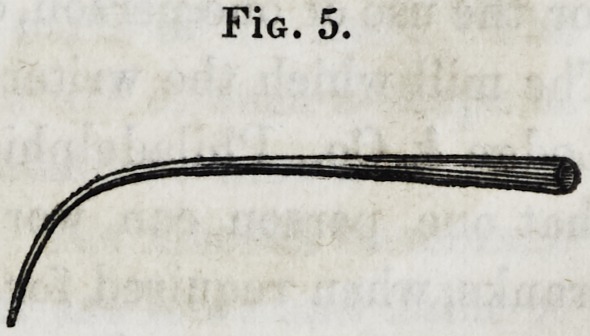


**Fig. 6. f6:**
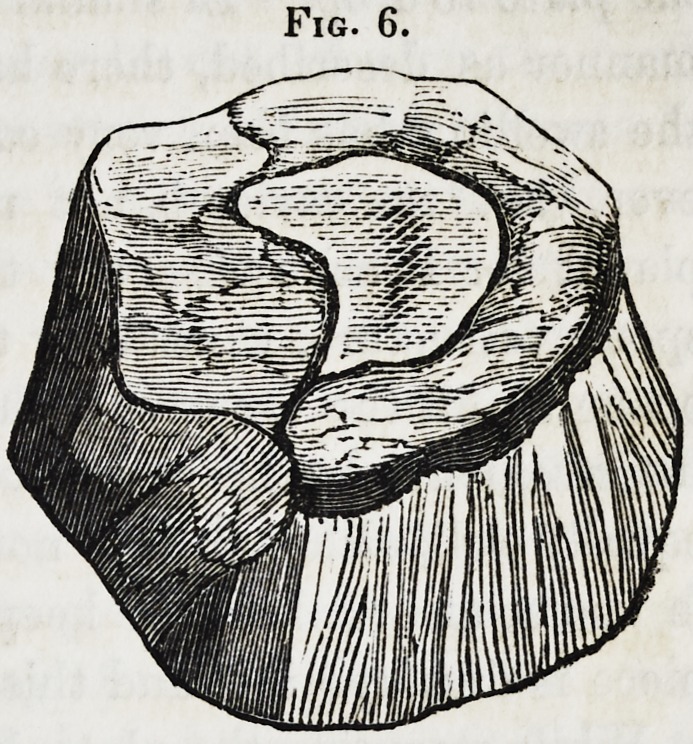


**Fig. 7. f7:**
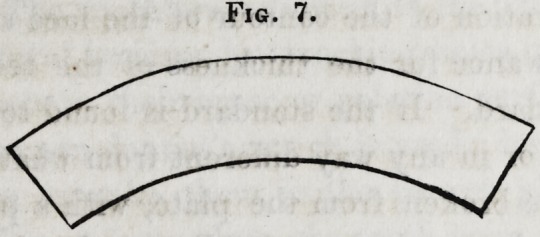


**Fig. 8. f8:**
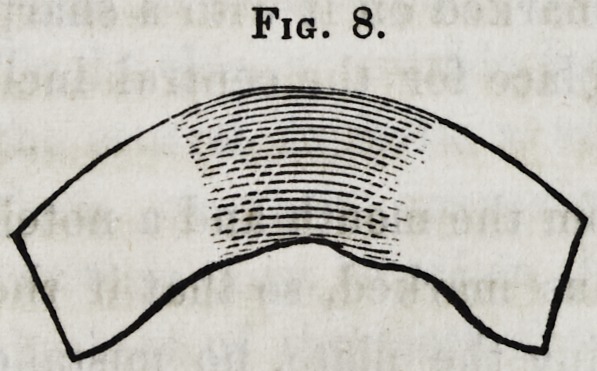


**Fig. 9. f9:**
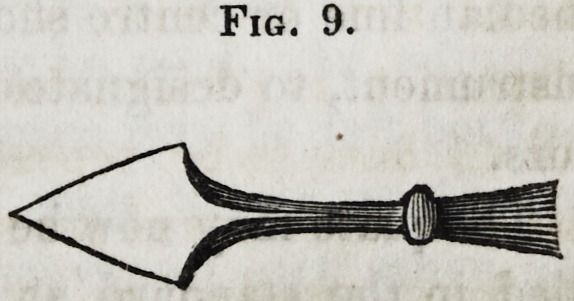


**Fig. 10. f10:**
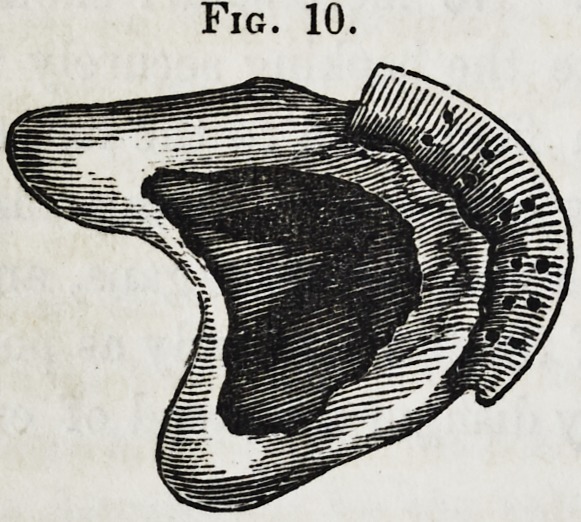


**Fig 11. f11:**
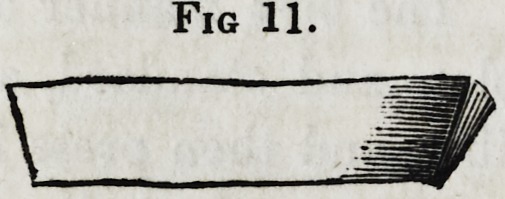


**Fig. 12. f12:**
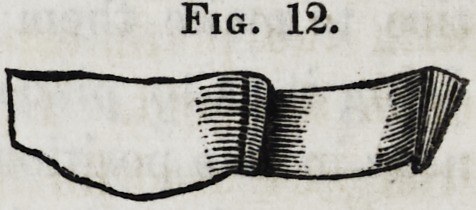


**Fig. 13. f13:**
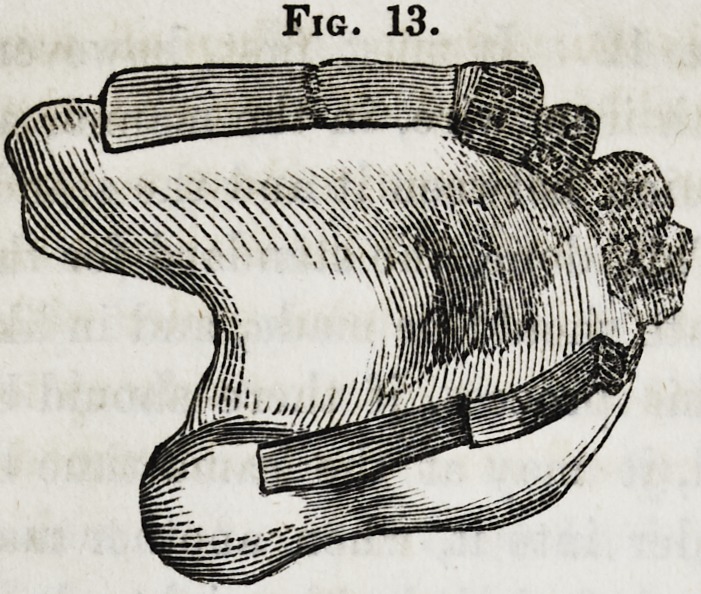


**Fig. 14. f14:**
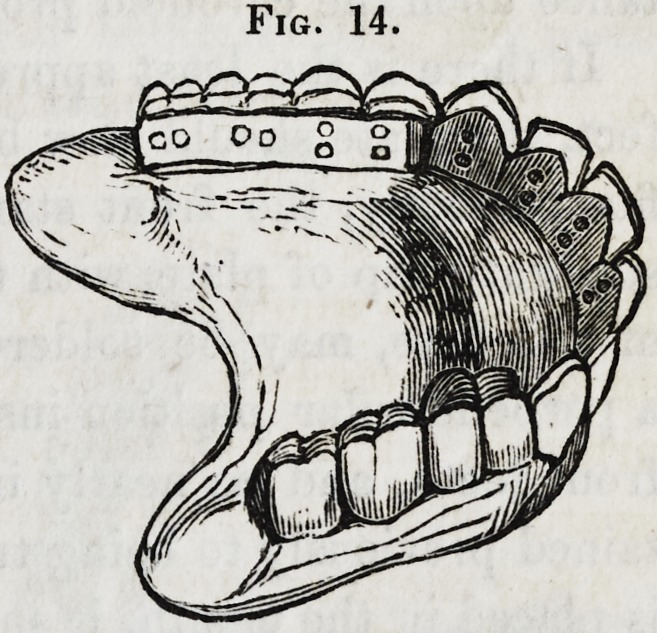


**Fig. 15. f15:**
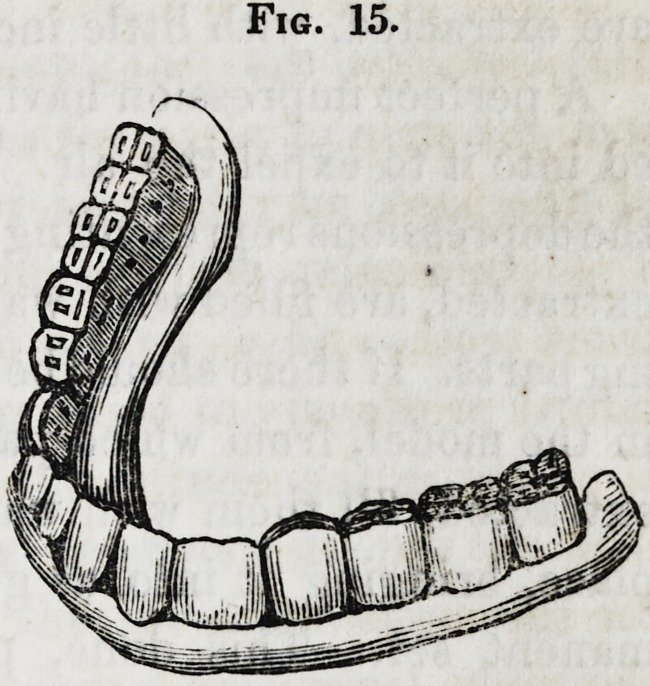


**Fig. 16. f16:**
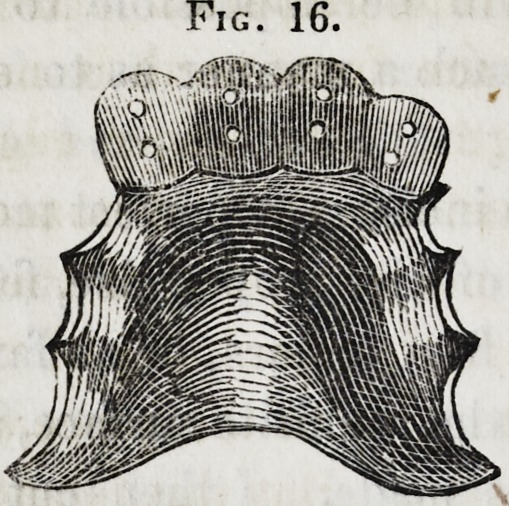


**Fig. 17. f17:**
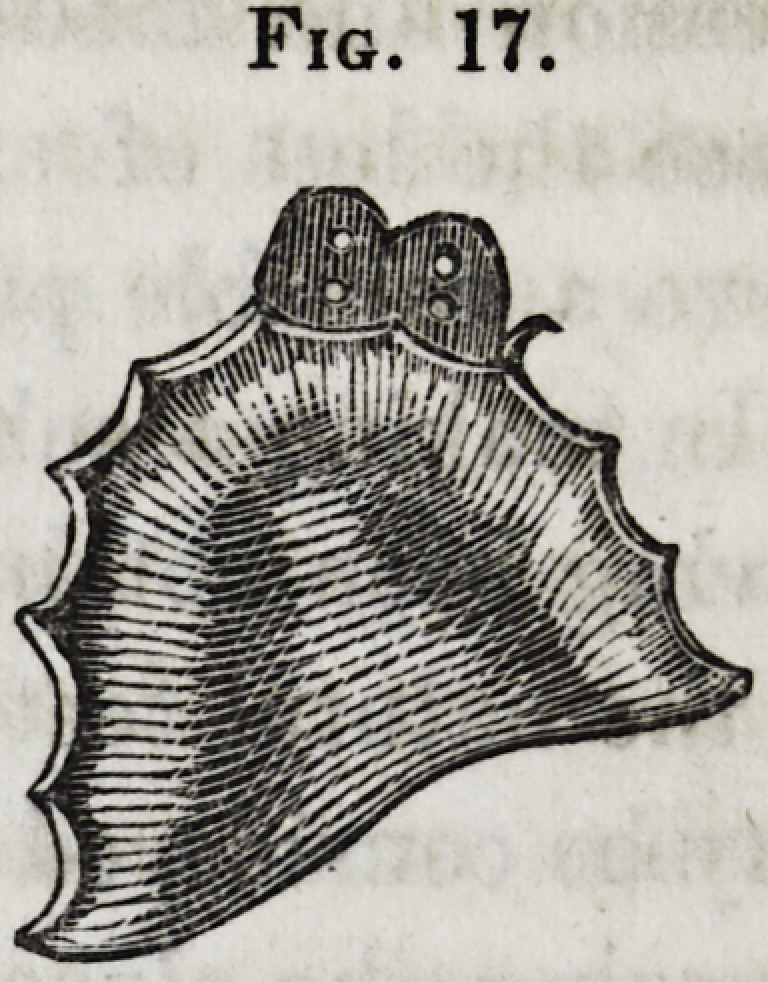


**Fig. 18. f18:**